# Timing and causes of death in severe COVID-19 patients

**DOI:** 10.1186/s13054-021-03639-w

**Published:** 2021-06-30

**Authors:** Charles de Roquetaillade, Swann Bredin, Jean-Baptiste Lascarrou, Thibaud Soumagne, Mariana Cojocaru, Benjamin Glenn Chousterman, Maxime Leclerc, Albin Gouhier, Gaël Piton, Frédéric Pène, Annabelle Stoclin, Jean-François Llitjos

**Affiliations:** 1 Department of Anesthesiology and Critical Care, Hôpital Lariboisière, FHU PROMICE, DMU Parabol, APHP. Nord, Paris, France; 2 Inserm U942 MASCOT, Paris, France; 3 Intensive Care Unit, Hôpital Cochin, Assistance Publique-Hôpitaux de Paris Centre, Paris, France; 4 Intensive Care Unit, Hôpital Hôtel-Dieu, Nantes, France; 5 Intensive Care Unit, Hôpital Jean Minjoz Hospital, Besançon, France; 6 Surgical Intensive Care Unit, Hôpital Cochin, Assistance Publique-Hôpitaux de Paris Centre, Paris, France; 7 Intensive Care Unit, Centre Hospitalier Mémorial France Etats-Unis, Saint-Lô, France; 8 Intensive Care Unit, Centre Hospitalier Intercommunal Alençon Mamers, Alençon, France; 9 Intensive Care Unit, Gustave Roussy, Université Paris-Saclay, Villejuif, France

**Keywords:** COVID-19, SARS-CoV-2, Intensive care unit, Mortality, Causes of death

## Abstract

**Background:**

Previous studies reporting the causes of death in patients with severe COVID-19 have provided conflicting results. The objective of this study was to describe the causes and timing of death in patients with severe COVID-19 admitted to the intensive care unit (ICU).

**Methods:**

We performed a retrospective study in eight ICUs across seven French hospitals. All consecutive adult patients (aged ≥ 18 years) admitted to the ICU with PCR-confirmed SARS-CoV-2 infection and acute respiratory failure were included in the analysis. The causes and timing of ICU deaths were reported based on medical records.

**Results:**

From March 1, 2020, to April 28, 287 patients were admitted to the ICU for SARS-CoV-2 related acute respiratory failure. Among them, 93 patients died in the ICU (32%). COVID-19-related multiple organ dysfunction syndrome (MODS) was the leading cause of death (37%). Secondary infection-related MODS accounted for 26% of ICU deaths, with a majority of ventilator-associated pneumonia. Refractory hypoxemia/pulmonary fibrosis was responsible for death in 19% of the cases. Fatal ischemic events (venous or arterial) occurred in 13% of the cases. The median time from ICU admission to death was 15 days (25th–75th IQR, 7–27 days). COVID-19-related MODS had a median time from ICU admission to death of 14 days (25th–75th IQR: 7–19 days), while only one death had occurred during the first 3 days since ICU admission.

**Conclusions:**

In our multicenter observational study, COVID-19-related MODS and secondary infections were the two leading causes of death, among severe COVID-19 patients admitted to the ICU.

## Introduction

The precise mortality rate of the most severe forms of SARS-CoV-2 infections that are admitted to the intensive care unit (ICU) varies among studies, ranging from 8.1 to 30% [1–3]. In addition to differences in patient characteristics at admission and heterogeneity in management, the absence of a description of cause of death limits their interpretation. Two recent single-center studies specifically reported the timing and causes of death in severe COVID-19 patients [4, 5]. Despite reporting respiratory failure as the major cause of death, these two studies differ by reporting either organ failure or syndrome-based approaches, thereby limiting comparison.

However, characterizing the timing of death in patients along with the cause seems to be essential for better understanding COVID-19 and to guide further research. Indeed, other than corticosteroids and interleukin-6 receptor antagonists, several interventional studies failed to improve the outcome of patients with severe COVID-19 despite a plausible scientific rationale [6, 7]. As in septic shock patients, this failure might reflect an important underlying heterogeneity in patients. Therefore, reporting and describing the precise causes and timing of death in severe COVID-19 patients allows recognition of such heterogeneity and urges to better determine which patients could benefit from immunomodulatory therapeutics.

Herein, we report a multicenter retrospective analysis of severe COVID-19 patients admitted to eight French ICUs with the aim of determining the causes and timing of death.

## Material and methods

### Study design and subjects

We performed a retrospective study in eight ICUs within seven French hospitals (Institut Gustave Roussy, Paris; Cochin Hospital (2 ICU), Paris; Hotel Dieu Hospital, Nantes; Jean Minjoz Hospital, Besançon; Center Hospitalier Intercommunal, Alençon; Center Hospitalier Mémorial, Saint-Lô and Lariboisière Hospital, Paris). The study included consecutive adult patients (aged ≥ 18 years) admitted to the ICU with a PCR-confirmed SARS-CoV-2 infection and acute respiratory failure, defined as the need for at least 6 L/min of supplemental oxygen. Demographic, clinical, laboratory, treatment, and outcome data were collected from electronic medical records using a standardized data collection form. This study was approved by the Research Ethics Commission of the Institut Gustave Roussy. The study was registered at the French National Commission on Informatics and Liberty and the French National Institute for Health Data.

### Definition of patients

Laboratory confirmation of COVID-19 was based on SARS-CoV-2 detection by real-time RT-PCR from nasal swabs or lower respiratory tract secretions. Severity at admission was assessed using the Simplified Acute Physiology Score 2 and Sequential Organ Failure Assessment scores. Patients were considered immunocompromised if one or more of the following conditions were observed in the patients: solid tumors with chemotherapy in the last 3 months or progressive metastatic diseases, hematologic malignancies, solid organ transplantation, HIV infection with or without AIDS, treatment with corticosteroids (> 3 months, at any dosage or ≥ 1 mg/kg prednisone equivalent per day for > 7 days), or treatment with other immunosuppressive drugs. Acute respiratory distress syndrome (ARDS) was diagnosed according to the Berlin definition [8]. Obesity was defined as a body mass index of > 30 kg/m^2^.

### ICU management

Patients were managed according to local standards of care at each center in the pandemic context of the SARS-CoV-2 outbreak. Patients developing ARDS received neuromuscular blockade, high PEEP levels, and prone positioning, according to international guidelines [8]. Extracorporeal membrane oxygenation (ECMO) was used as salvage therapy in cases of persistent refractory hypoxemia, depending on center expertise and availability. In each center, end-of-life (EOL) decisions to withhold or withdraw life support were taken on collectively when maintenance or increase in life-sustaining therapies was considered futile by all staff participants, and death would irremediably occur in a short-term manner.

### Analysis and characteristics of death in COVID-19 patients

The cause of in-hospital death was defined as the syndrome responsible for fatality rather than organ failure. Disorders responsible for death were determined by analysis of the medical reports by two independent senior intensivists who were unaware of the first adjudication, with the help of an abstract paper document. Cases with discordant classification were adjudicated by consensus, with a check on inter-observer reliability. Causes of death were determined using a pre-specified set of syndromes defined a priori, based on clinical experience and a review of the existing literature. COVID-19-related multiple organ dysfunction syndrome (MODS) is defined as the dysfunction of two or more organs, including pulmonary, coagulation, cardiac, neurological, renal, hepatic, and gastrointestinal manifestations, that were not preexistent before SARS-CoV-2 infection [9]. ICU-acquired infections were defined as probable or definite according to confirmed microbiological assessment or strong clinical suspicion without microbiological assessment. The definition of ICU-acquired pneumonia was based on French guidelines [10]. We also considered secondary infection-related MODS as a cause of death, defined according to (1) the existence of a secondary infection, and (2) a compatible clinical course with clinical deterioration occurring after a transient improvement following admission. Refractory hypoxemia was defined as a PaO_2_ < 60 mmHg for more than 1 h while receiving a FiO_2_ of 1.0 [11], that led to intractable hypoxemia and/or hypercapnia. Fatal mesenteric or limb ischemia leading to MODS, fatal myocardial infarction, pulmonary embolism leading to cardiac arrest, or major stroke accounted for fatal thromboembolic events. As EOL decision making and care is a result of the severity of the underlying process, rather than a cause itself, we analyzed EOL decision as an outcome rather than a cause of death.

### Statistical analysis

For the descriptive analysis, continuous variables were expressed as medians (interquartile range), and categorical variables as numbers (percentages). Comparisons were performed using the Fisher exact test or *χ*^2^ test for categorical data, and the Kruskal–Wallis or Wilcoxon test for continuous data. The time for admission to ICU death was classified according to the causes of death and compared using one-way ANOVA with Dunn’s multiple comparison test. To identify independent predictors of hospital mortality, characteristics associated with *p* values less 0.1 by univariate analysis, or deemed clinically relevant were included in a multivariable logistic regression model with backward selection. Non-log-linear continuous variables were dichotomized. All analyses were performed using R version 3.6 (R project, Vienna).

## Results

### Patients’ characteristics

From March 1, 2020, to April 28, 287 patients were admitted to the ICU for SARS-CoV-2 related acute respiratory failure. Their main characteristics are listed in Table 1. The patients were mostly male (*n* = 232, 80.8%), with a median age of 63 (54–71) years. Arterial hypertension and obesity were the prominent comorbid conditions. Most of them were mechanically ventilated (91%) with a median duration of 19 days (25th–75th IQR: 13–28 days), and approximately two-thirds underwent prone positioning. Rescue extracorporeal membrane oxygenation (ECMO) was administered to 25 patients (8%).

**Table 1:** Characteristics of the patients

	All patients (*n* = 287)	Survivors (*n* = 194)	Deceased in ICU (*n* = 93)	*p*
Age, years	63 [54–71]	61.00 [52–69]	68.00 [61–75]	< 0.01
Female gender	55 (19.2)	40 (20.6)	15 (16.1)	0.46
Body mass index	28 [25–32]	28 [25–32]	28 [25–31]	0.94
*Comorbid conditions*				
Obesity	168 (61.3)	116 (62.7)	52 (58.4)	0.58
Arterial hypertension	137 (47.7)	89 (45.9)	48 (51.6)	0.43
Diabetes mellitus	84 (29.3)	54 (27.8)	30 (32.3)	0.53
Tobacco use	38 (13.2)	26 (13.4)	12 (12.9)	1
COPD	34 (11.8)	22 (11.3)	12 (12.9)	0.85
Chronic kidney disease	22 (7.7)	11 (5.7)	11 (11.8)	0.11
Cirrhosis	2 (0.7)	1 (0.5)	1 (1.1)	1
*Characteristics on ICU admission*				
SAPS2, points	59 [29–53]	36 [28–46]	47 [38–63]	< 0.01
SOFA, points	6 [4–9]	5 [3–8]	8 [5–10]	< 0.01
Initial P/F ratio	103 [76–150]	113 [80–150]	86 [70–124]	< 0.01
Interval from symptom onset to ICU admission	7 [4–10]	7 [4–10]	7 [4–10]	0.93
*ICU management*				
Mechanical ventilation	262 (91.3)	170 (87.6)	92 (98.9)	< 0.01
Norepinephrine	217 (75.9)	130 (67.4)	87 (93.5)	< 0.01
Neuromuscular blockade	244 (85.0)	154 (79.4)	90 (96.8)	< 0.01
Prone positioning	194 (67.6)	113 (58.2)	81 (87.1)	< 0.01
Extracorporeal membrane oxygenation	25 (8.7)	11 (6.4)	14 (15.7)	0.03
Renal replacement therapy	82 (28.6)	38 (19.6)	44 (47.3)	< 0.01
*Specific treatment*				
Hydroxychloroquine	84 (29.3)	55 (28.5)	29 (31.5)	0.70
Azithromycin	75 (26.1)	48 (24.9)	27 (29.3)	0.51
Lopinavir/ritonavir	61 (21.3)	33 (17.1)	28 (30.4)	0.02
Corticosteroids	55 (19.2)	31 (18.0)	24 (27.3)	0.12
Remdesivir	10 (3.4)	9 (4.7)	1 (1.1)	0.23
Tocilizumab	8 (2.8)	3 (1.6)	5 (5.4)	0.14
*Outcomes*				
Length of stay, days	19 [10–30]	19 [12–31]	15 [7–27]	0.04
Duration of mechanical ventilation, days	16 [8–28]	16 [9–28]	15 [7–27]	0.75
Secondary infection	151 (53.0)	97 (50.3)	54 (58.7)	0.23
End-of-life decision	42 (14.6)	0 (0)	42 (45.2)	< 0.01

ICU, Intensive care unit; COPD, chronic obstructive pulmonary disease; SAPS, simplified acute physiology *score*; SOFA, Sepsis-related Organ Failure Assessment

### Causes and timing of death

COVID-19-related MODS was the leading cause of death (37%, *n* = 35/93). ICU death resulted from secondary infection-related MODS in 26% of patients (*n* = 24/93), with a majority of ICU-acquired pneumonia (ICU-AP). Refractory hypoxemia was responsible for death among patients with severe COVID-19 in 19% of the cases (*n* = 18/93). Fatal ischemic events were responsible for ICU death in 13% of patients (seven cases of pulmonary embolism, two strokes, two mesenteric ischemia, and one myocardial infarction). The inter-observer reliability was 93%, with discordant classification that required adjudication by consensus in seven cases. Patient characteristics as per the cause of death are depicted in Table 2. The absence of neuromuscular blockade was associated with death from refractory hypoxemia/pulmonary fibrosis (*p* < 0.01). EOL decisions were recorded in 42 deceased patients’ files (45.1%) and their deaths were mostly associated with refractory hypoxemia/pulmonary fibrosis (*p* < 0.01), while secondary infection-related MODS was mostly associated with the absence of EOL decisions (*p* < 0.01) (Table 3).

**Table 2:** Baseline characteristics and outcome of Covid-19 patients who died in ICU stratified according to cause of death

	COVID-19-related MODS (*n* = 35)	Secondary infection-related MODS (*n* = 24)	Refractory hypoxemia (*n* = 18)	Fatal ischemic event (*n* = 13)	Others* (*n* = 3)	*p*
Age, years	71 [63–76]	66 [62–71]	670 [62–77]	61 [56–74]	60 [51–64]	0.14
Female gender	7 (20)	3 (12.5)	2 (11.1)	3 (23.1)	0 (0)	0.74
Body mass index	29 [26–32]	28 [26–30]	26 [24–29]	28 [27–31]	32 [28–32]	0.52
*Comorbid conditions*						
Obesity	21 (63.6)	11 (45.8)	10 (62.5)	8 (61.5)	2 (66.7)	0.70
Arterial hypertension	18 (51.4)	11 (45.8)	11 (61.1)	7 (53.8)	1 (33.3)	0.84
Diabetes mellitus	13 (37.1)	8 (33.3)	5 (27.8)	4 (30.8)	0 (0)	0.74
Tobacco use	6 (17.1)	3 (12.5)	2 (11.1)	1 (7.7)	0 (0)	0.85
COPD	3 (8.6)	6 (25.0)	2 (11.1)	1 (7.7)	0 (0)	0.34
Chronic kidney disease	5 (14.3)	3 (12.5)	3 (16.7)	0 (0)	0 (0)	0.60
Cirrhosis	0 (0)	1 (4.2)	0 (0)	0 (0)	0 (0)	0.57
*Characteristics on ICU admission*						
SAPS2, points	47 [38–63]	47 [43–60]	50 [38–63]	42 [27–65]	32 [26–58]	0.78
SOFA, points	8 [5–11]	8 [6–11]	8 [5–9]	8 [6–10]	9 [6–9]	0.85
Initial P/F ratio	100 [70–147]	80 [72–87]	79 [65–120]	106 [70–190]	98 [84–144]	0.58
Interval from symptom onset to ICU admission	7 [4–9]	7 [5–9]	8 [5–10]	10 [7–10]	8 [6–9]	0.66
*ICU management*						
Mechanical ventilation	35 (100)	23 (95.8)	18 (100)	13 (100)	3 (100)	0.57
Norepinephrine	35 (100)	23 (95.8)	15 (83.3)	11 (84.6)	3 (100)	0.11
Neuromuscular blockade	35 (100)	24 (100)	15 (83.3)	13 (100)	3 (100)	0.01
Prone positioning	31 (88.6)	22 (91.7)	14 (77.8)	11 (84.6)	3 (100)	0.66
Extracorporeal membrane oxygenation	3 (8.8)	5 (22.7)	2 (11.1)	3 (25)	1 (33.3)	0.43
Renal replacement therapy	18 (51.4)	16 (66.7)	4 (22.2)	5 (38.5)	1 (33.3)	0.06
*Specific treatment*						
Hydroxychloroquine	12 (34.3)	10 (41.7)	3 (17.6)	2 (15.4)	2 (66.7)	0.19
Azithromycin	10 (28.6)	11 (45.8)	3 (17.6)	2 (15.4)	1 (33.3)	0.24
Lopinavir/ritonavir	13 (37.1)	6 (25)	4 (23.5)	3 (23.1)	2 (66.7)	0.46
Corticosteroids	12 (35.3)	6 (27.3)	2 (11.8)	3 (25)	1 (33.3)	0.52
Tocilizumab	2 (5.7)	2 (8.3)	1 (5.9)	0 (0)	0 (0)	0.86
Remdesivir	0 (0)	1 (4.2)	0 (0)	0 (0)	0 (0)	0.57
*Outcomes*						
Length of stay, days	14 [7–24]	20 [9–30]	12 [9–27]	16 [6–27]	31 [18–32]	0.60
End-of-life decisions	17 (48.6)	4 (16.7)	15 (83.3)	4 (30.8)	2 (66.7)	< 0.01

^*^One patient died from iatrogenic event, one patient died from cardiac arrest of unknown origin, and one patient died from invasive aspergillosis. ICU: Intensive care unit; COPD: chronic obstructive pulmonary disease; SAPS: simplified acute physiology *score*; SOFA: Sepsis-related Organ Failure Assessment

**Table 3:** Comparison of causes of death between patients with end-of-life decision and others

	All *n* = 93	No EOL decision *n* = 51	EOL decision *n* = 42	*p*
Length-of-stay, days	15 [7–27]	14 [7–26]	17 [9–27]	0.55
*Cause of death*				
Covid-19-related MODS	35 (37.6)	18 (35.3)	17 (40.5)	0.76
Secondary infection-related MODS	24 (25.8)	20 (39.2)	4 (9.5)	< 0.01
Refractory hypoxemia	18 (19.4)	3 (5.9)	15 (35.7)	< 0.01
Fatal ischemic event	13 (14)	9 (17.6)	4 (9.5)	0.41
Other	3 (3.2)	1 (2)	2 (4.8)	0.86

EOL: End-of-life; MODS: multiple organ dysfunction syndrome

**Fig. 1:**
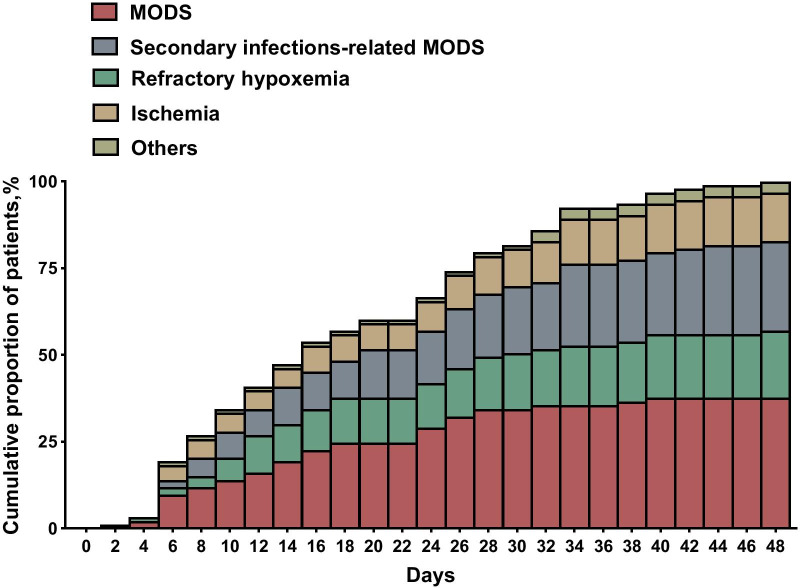
Cumulative incidence of ICU-mortality during hospitalization. ICU: Intensive Care Unit, MODS: multiple organ dysfunction syndrome

The median time from ICU admission to death was 15 days (25th–75th IQR, 7–27 days). COVID-19-related MODS had a median time from ICU admission to death of 14 days (25th–75th IQR: 7–19 days). One patient had died during the first three days after ICU admission due to COVID-related MOF. We found no statistical differences in the time from admission to ICU death between the different causes of death (Table 2). Time to death was not different between patients with EOL and those without EOL (14 [7–26] vs. 17 [9–27] days, *p* = 0.55) (Table 3). The distribution of deaths according to the time from admission is depicted in Fig. 1.

## Discussion

In our multicenter observational study, COVID-19-related MODS and secondary infections were determined to be the two main causes of death in severe COVID-19 patients admitted to the ICU.

Our investigation into the timing and causes of death in severe COVID-19 patients stands out, irrespective of previous studies in the same topic currently in the literature [4, 5], due to the following distinct points. First, this is the first multi-center study, albeit in the same country. Second, in contrast to Ketcham et al., who studied organ dysfunction as the main cause of death, we reported causes of disease processes. Finally, regarding the classification of diseases, our study is the first to assess the burden of secondary infection-related death, which is an emerging threat during the massive utilization of corticosteroids and immunomodulatory drugs among COVID-19 patients [12]. Prior studies report high proportions of septic shock as a cause of death in patients with severe COVID-19, ranging from 15 to 27%. According to the SEPSIS-3 definition of septic shock [13], the lactate level must be higher than 2 mmol/L. However, it is noteworthy that lactate values in septic shock patients mostly do not exceed this threshold, as reported in a large cohort of 4,244 severe COVID-19 patients [2]. Considering this, we reported the existence of MODS rather than septic shock to avoid the gathering of several different etiologies under the identity of septic shock. Moreover, studies reporting causes of death in patients with SARS-CoV-2 infection admitted to the ICU report either a striking 48% mortality rate [5] or no data regarding the in-ICU mortality rate [4], therefore limiting the external validity of these results.

Although understanding the causes of mortality is of major interest, identifying the precise pathway to death faces several constraints. First, assigning a single cause of death is complex and their definitions may vary among practitioners. Therefore, in our study, all medical reports were blindly reviewed by two independent senior intensivists. Whether causal mediation analysis could have permitted precise causes of death, the retrospective design of the study without systematic biological sampling does not allow for such analysis. However, we assessed the variation in the proportion of each cause of death over time. We hypothesize that if there is a selection bias in our study, there is no reason that it would not have varied over time. Thus, since the proportion of each cause of death remains constant over time, the possibility of such a bias remains minimal. Second, several diseases may be deeply intertwined and lead to death, especially after a protracted period of ICU stay, which is associated with the occurrence of ICU-acquired complications. Thus, the attributable mortality of each potential cause of death remains highly debated, especially in severe COVID-19 patients [14]. Autopsy findings and histopathological postmortem evidence are therefore crucial for improving our understanding of severe COVID-19, especially in distinguishing the exact cause of death from other contributing factors. A recent review focusing on postmortem examinations in COVID-19 patients reported pulmonary embolism as a major cause of death in COVID-19, with a high prevalence of peripheral deep venous thromboembolism [15]. These observations are in line with several studies reporting both venous and arterial thrombotic events as common in severe COVID-19 patients [16–18]. Consequently, one can hypothesize that the high incidence of COVID-19-related MODS might rely on diffuse thromboembolic complications.

Unlike what is observed in severe COVID-19 patients in this study, early mortality is high in severe bacterial or viral pneumonia. In septic shock, approximately one-third of patients die during the first 72 h, with a vast majority being primary infection-related MODS [19]. Early mortality is also high in severe viral pneumonia, with the identification of bacterial co-infection as a major cause of death [20]. This early mortality has been ascribed to the existence of an overwhelming inflammation at the initial stages of septic shock, including the overexpression of pro-inflammatory cytokines such as IL-6, IL-12, and TNFα [21]. In line with our results, a recent study reported IL-6 serum levels to be 27-times lower in COVID-19 patients than in septic shock patients, therefore questioning the existence of a cytokine storm in COVID-19 [22]. Once again, the in-hospital course of COVID-19 patients admitted to the ICU is different, with a low reported rate of bacterial co-infection at admission as compared to other causes of viral pneumonitis [23]. However, recent studies have reported that patients with SARS-CoV-2 infection are at higher risk for ICU-acquired pneumonia [24, 25] as compared with other causes of pneumonitis, with some data suggesting a significant association between increased mortality and ICU-acquired pneumonia in severe COVID-19 patients [25]. However, precise evaluation of the attributable mortality, defined as the percentage of deaths that would not have occurred without infection, is complex for ICU-acquired pneumonia and requires the use of competing risk statistical models. Hence, recent studies have found little impact of ICU-acquired infections on ICU mortality [26, 27]. Therefore, our results urge the reappraisal of effects of ICU-acquired pneumonia on mortality in patients with severe COVID-19.

The EOL decision accounted for 45% of patients among deceased COVID-19 patients. Ethical issues in the ICU have been a challenging point in the COVID-19 pandemic context for two main reasons: 1) the higher mortality rate in the elderly and frail patients and 2) the shortage of medical resources [28]. Therefore, the high rate of life-sustaining therapy discontinuation could reflect the existence of unusual external constraints [29]. However, two points argue against this assertion: First, such a proportion has been previously reported, with EOL decision as the main cause of death in septic shock patients [19]. Second, the median time from admission to death in patients with life-sustaining therapy discontinuation is 16 days, which is higher than the time usually reported in the literature [30]. Finally, we report no difference in time to death between patients with and without EOL decisions, which suggests that withdrawal of care has been decided in a non-emergency context.

This study had several limitations. First, as stated by a recent publication, causal inferences from observational data are one of the main problems [31]. Whether our assessment of inter-observer reliability served to solely decrease the risks of interpretation bias, it could only be verified by further studies considering to perform causal mediation analysis using biomarkers. This approach was not possible in our current study due to the lack of longitudinal systematic blood sampling in our cohort. Another possibility could be to perform a competitive survival analysis. However, given the several intertwined competing factors and the sample size of our cohort, this approach was not possible. Second, the multicenter design is associated with differences in diagnosis procedures, and the determination of causes of death was left at the discretion of the physician in-charge. Third, the standard of care has evolved since the outbreak of the COVID-19 pandemic and might therefore limit the external validity of our results. Of note, published data on the topic are conflicting, some suggesting similar mortality between the different periods [32] or an increase over time of the risk-adjusted survival to hospital discharge [33]. Notably, the recent emergence of SARS-CoV-2 variants is reported to be associated with higher mortality rates [34]. However, data concerning the causes of mortality related to these emerging variants are missing and require further investigation. Finally, we focused our analysis on in-ICU death, therefore setting aside other causes of death that may rely on long-term effects of SARS-Cov-2 infection.

## Conclusion

We identified COVID-19-related MODS and secondary infections as the two main causes of death in severe COVID-19 patients admitted to the ICU, with a vast majority occurring after two weeks. These results urge to continue attempts to better understand the pathophysiology of this disease and to develop uniform diagnostic strategies.

## Declarations

### Availability of data and materials

All data supporting the results will be made available on motivated request to the corresponding author.
